# Supplementary material for: Digitizing mass spectrometry data to explore the chemical diversity and distribution of marine cyanobacteria and algae
Source: eLife. 2017 May 11;6:e24214. doi: 10.7554/eLife.24214 (PMC5441867; doi:10.7554/eLife.24214)
Supplement: Supplementary file 2. — DOI: http://dx.doi.org/10.7554/eLife.24214.016 [file elife-24214-supp2.docx]

**Supplementary file 2: GNPS molecular networking MS/MS based identification of molecules and molecular families.**

| **#** | **NP** | **Structure** | **Ref** | **Molecular family** |
| --- | --- | --- | --- | --- |
| 1 | Apratoxin B | 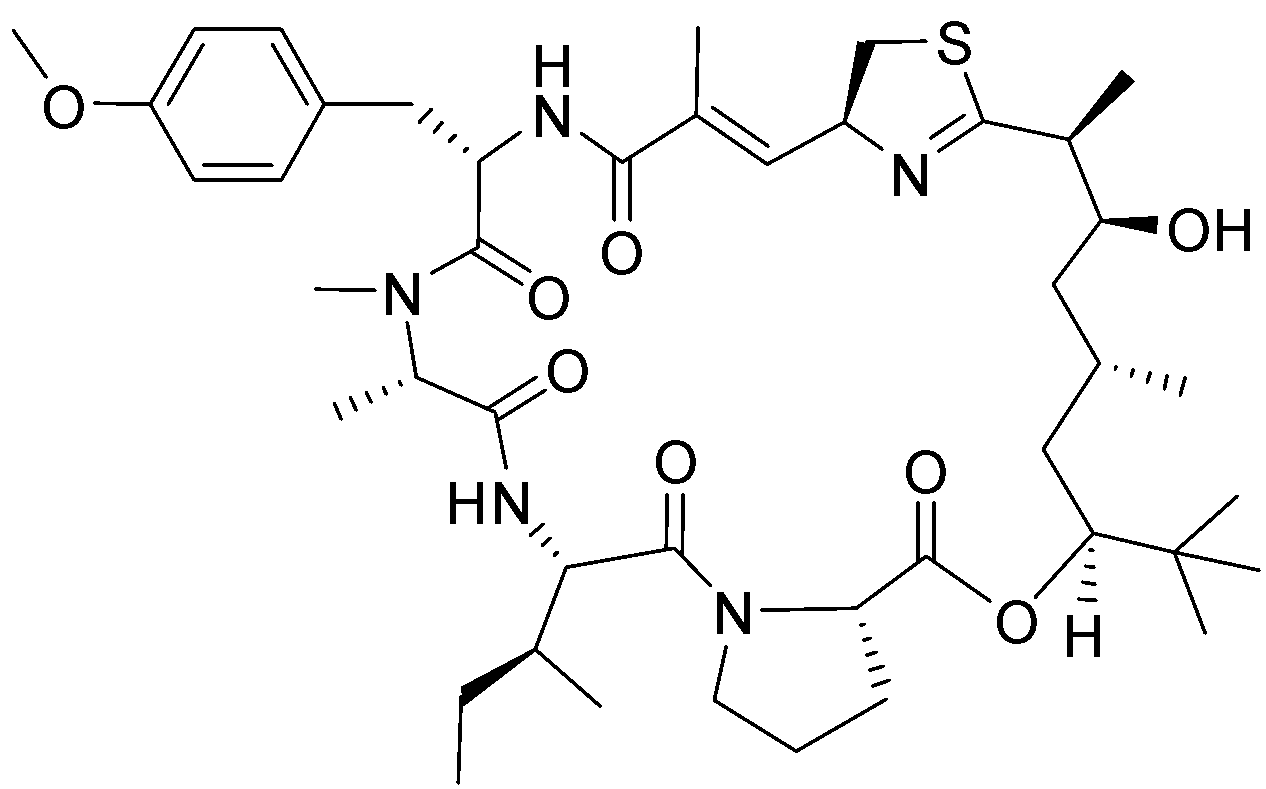 | ([Luesch, et al., 2002](#_z337ya)) | 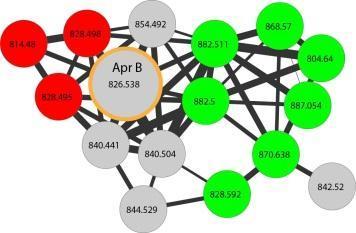 |
| 2 | Barbamide | 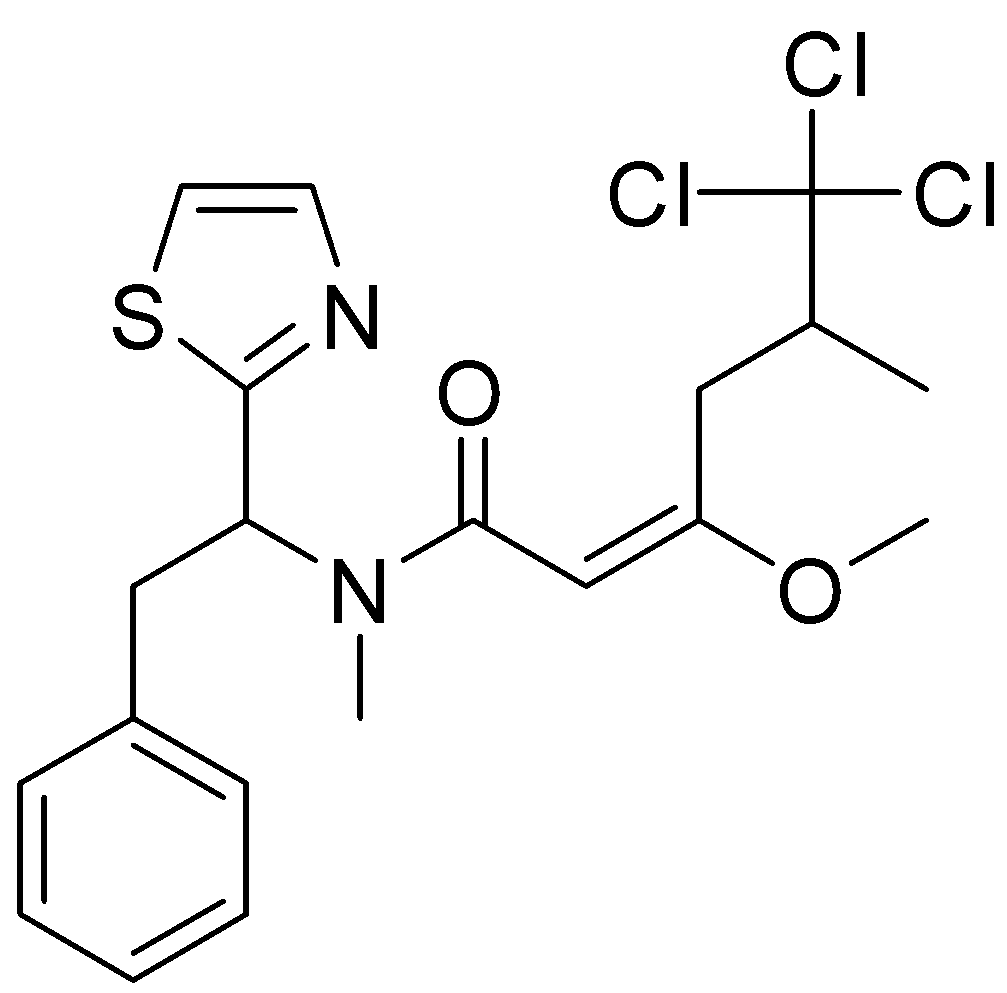 | ([Orjala and Gerwick, 1996](#_2xcytpi)) | 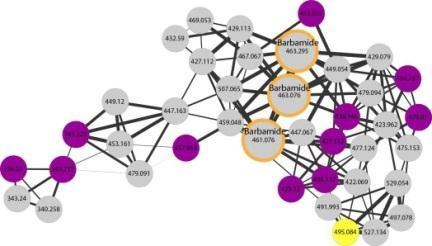 |
| 3 | Bouillonamide B | 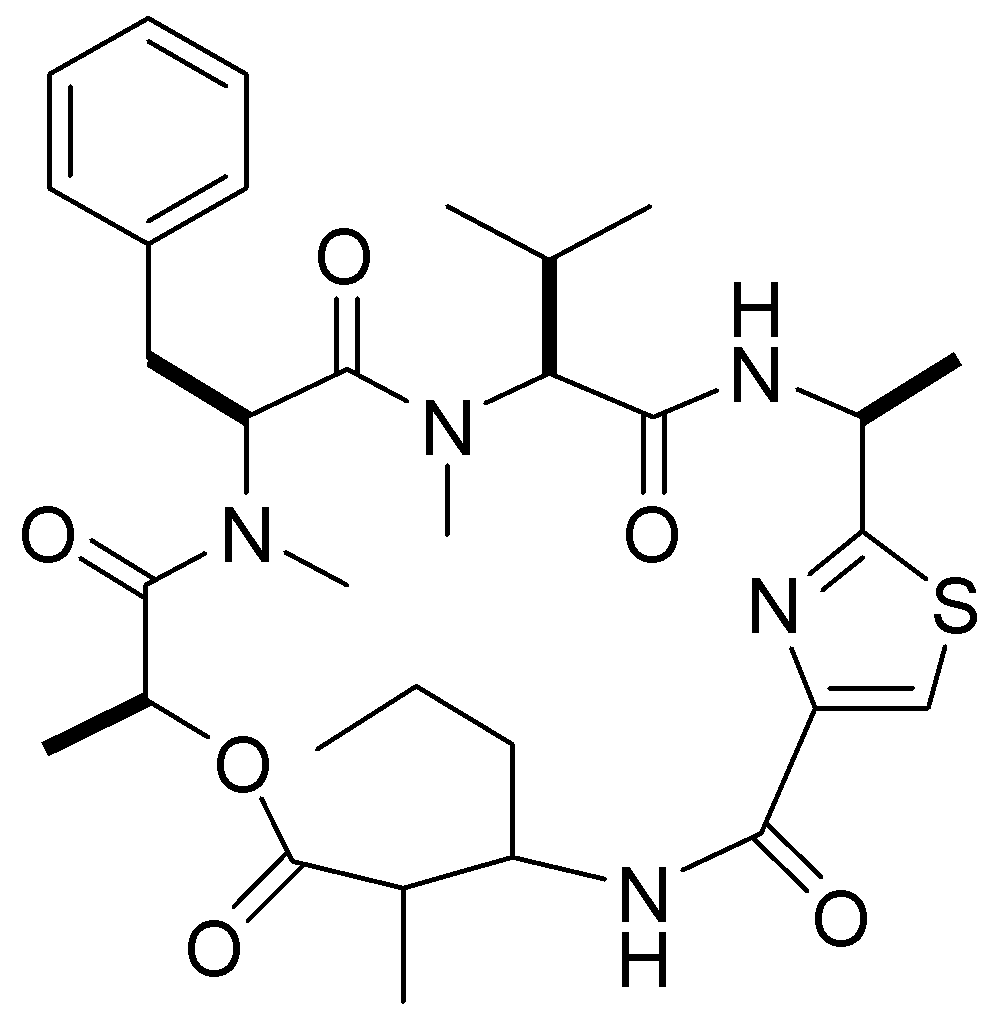 | ([Tan, et al., 2013](#_49x2ik5)) | 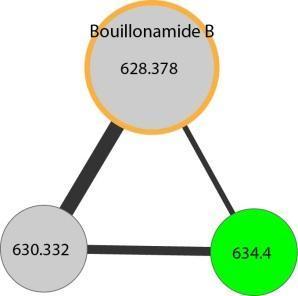 |
| 4 | Carmabin A | 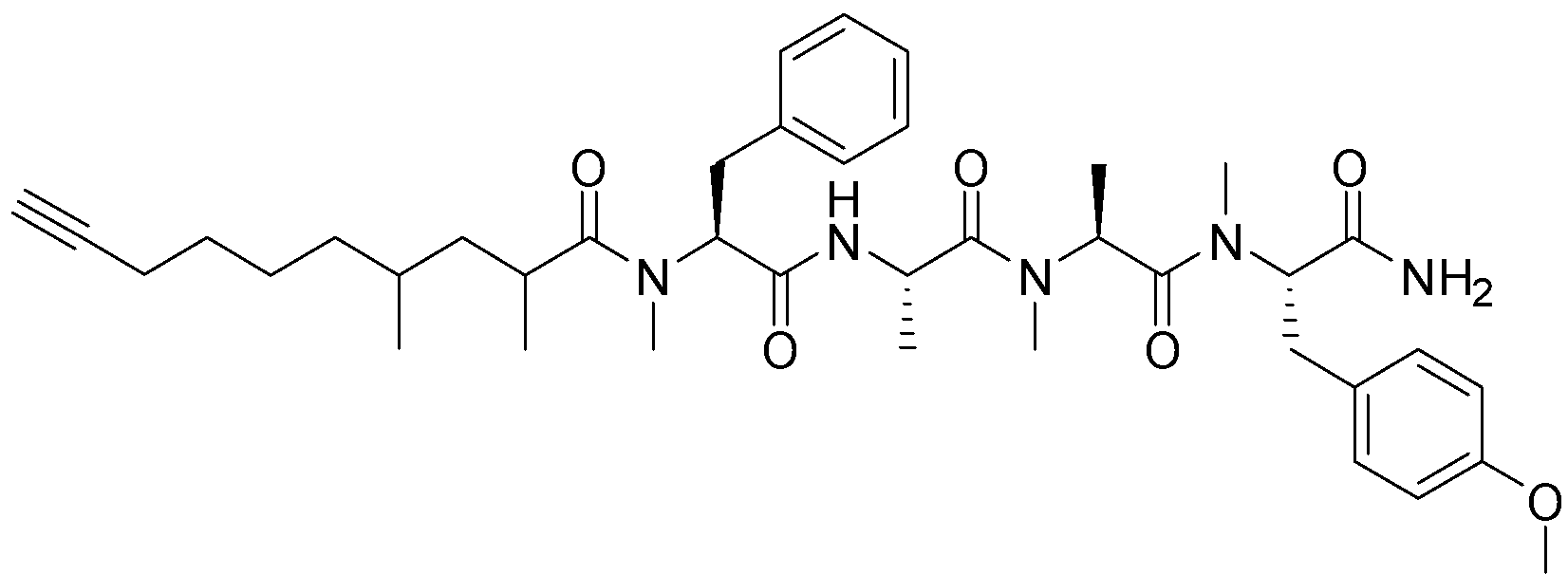 | ([Hooper, et al., 1998](#_2jxsxqh)) | 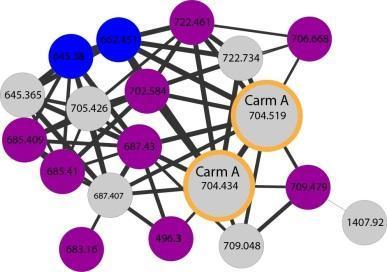 |
| 5 | Curacin A |  | ([Blokhin, et al., 1995](#_tyjcwt)) | 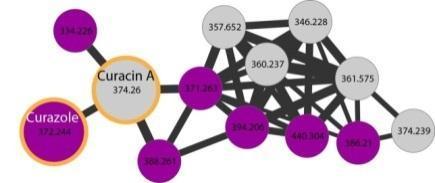 |
| 6 | Curazole |  | ([Esquenazi, et al., 2008](#_17dp8vu)) |  |
| 7 | Cyanolide A | 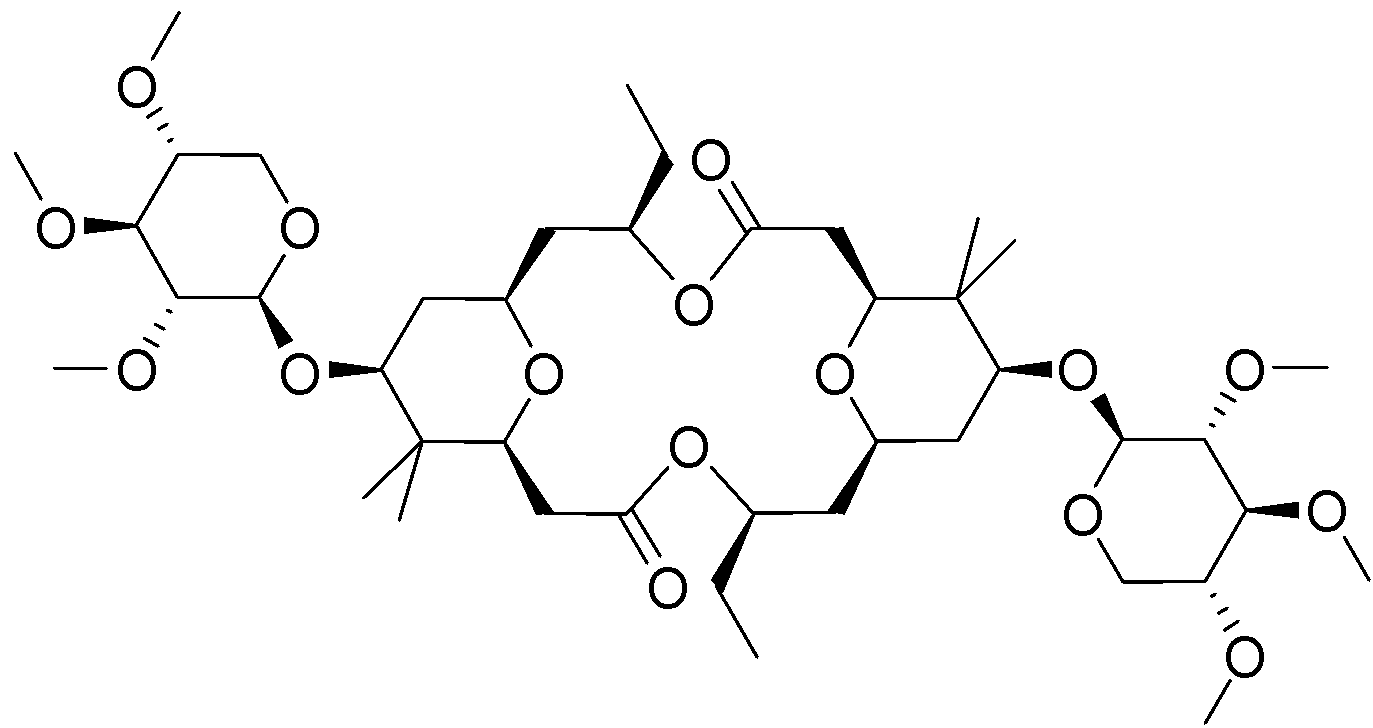 | ([Pereira, et al., 2010](#_qsh70q)) | 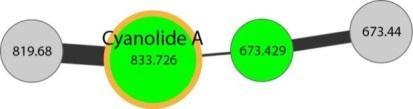 |
| 8 | Dolastatin 10 | 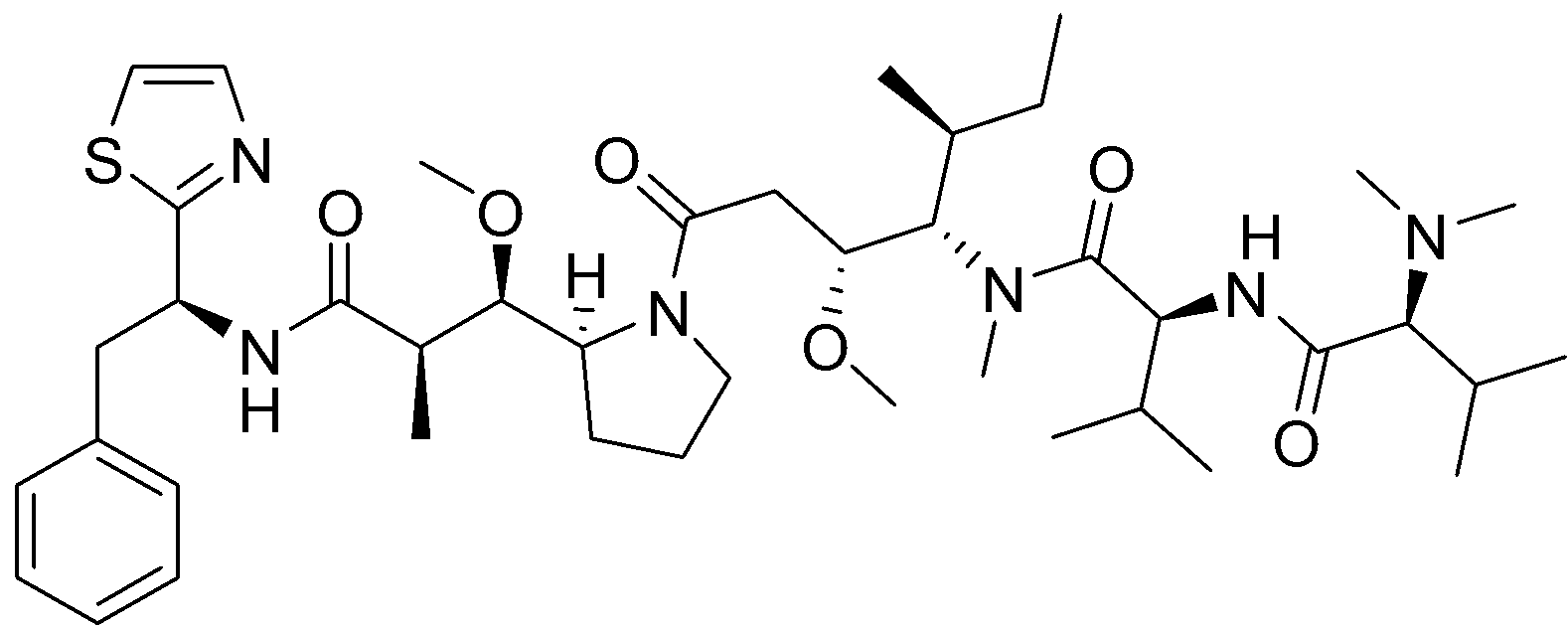 | ([Bai, et al., 1990](#_2et92p0)) | 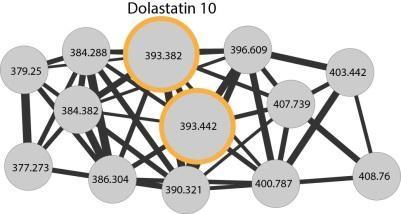 |
| 9 | Dolastatin 12 | 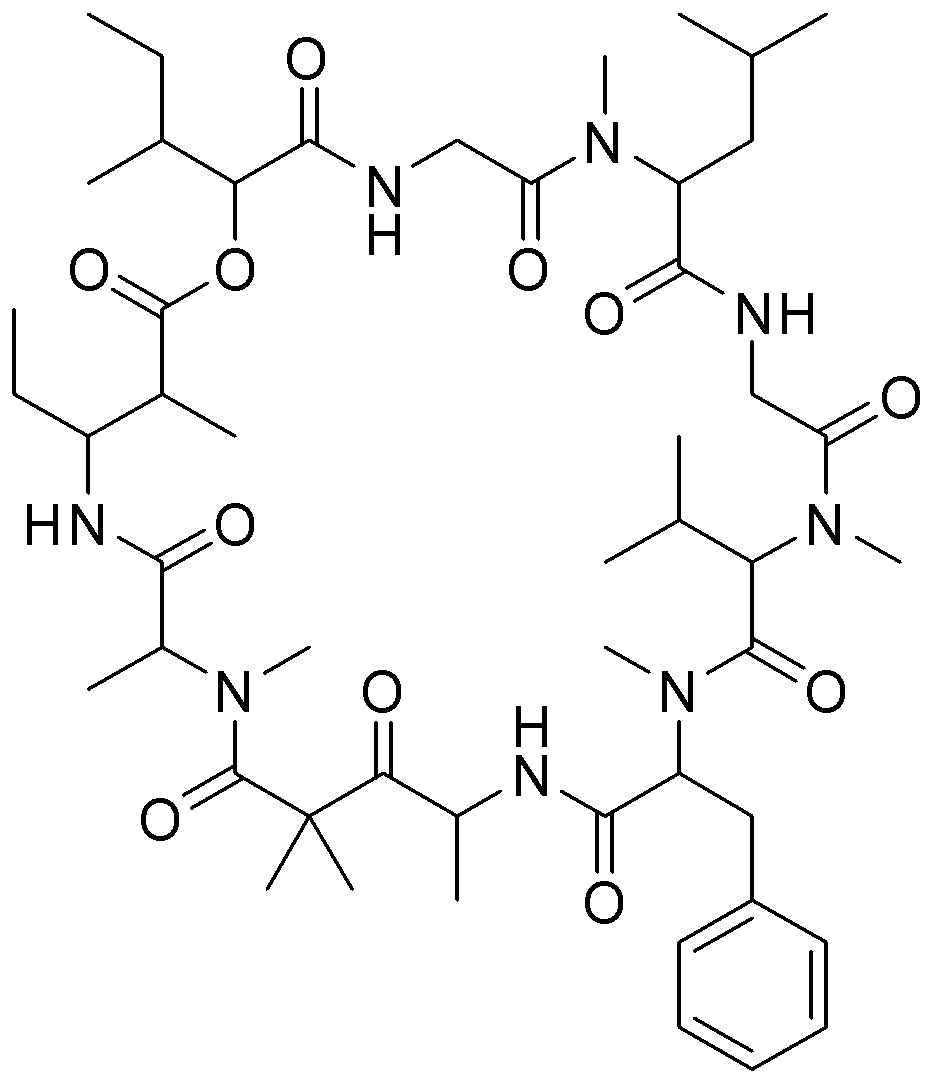 | ([Harrigan, et al., 1998](#_44sinio)) | 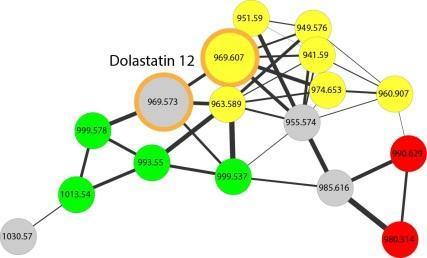 |
| 10 | Ecklonialactone A | 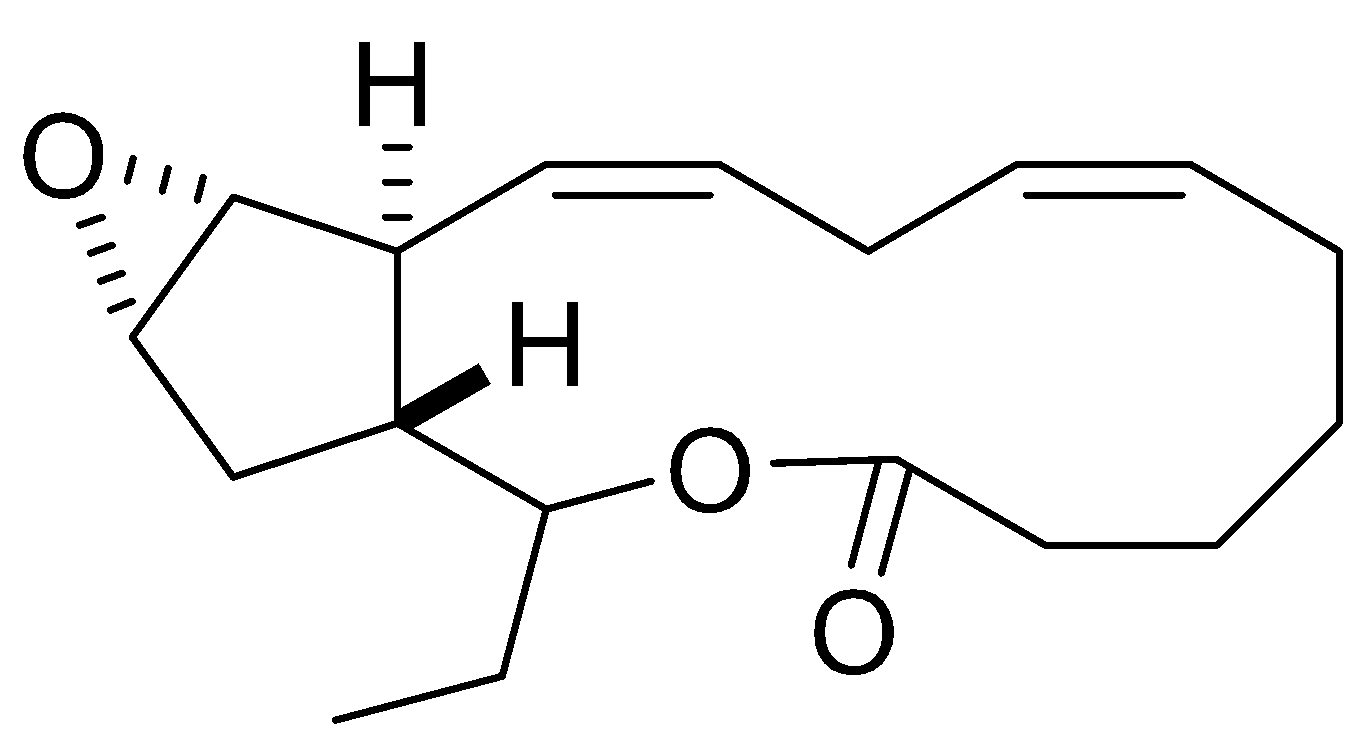 | ([Todd, et al., 1994](#_147n2zr)) | 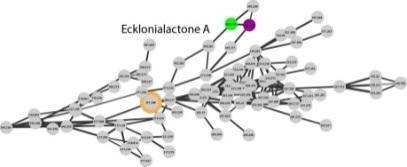 |
| 11 | Hectochlorin | 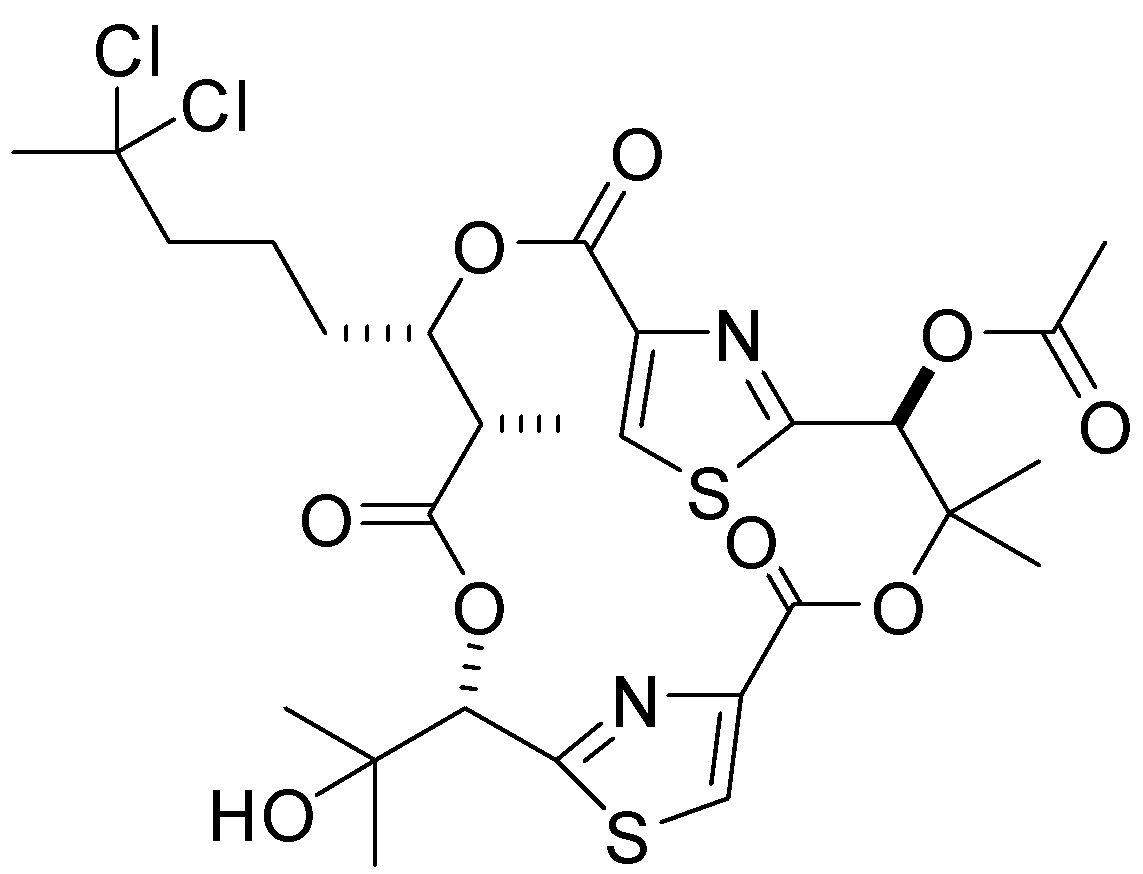 | ([Marquez, et al., 2002](#_3j2qqm3)) | 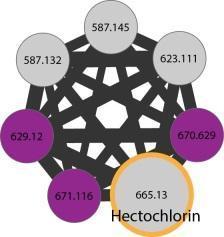 |
| 12 | Hoiamide A  [M+H]+ | 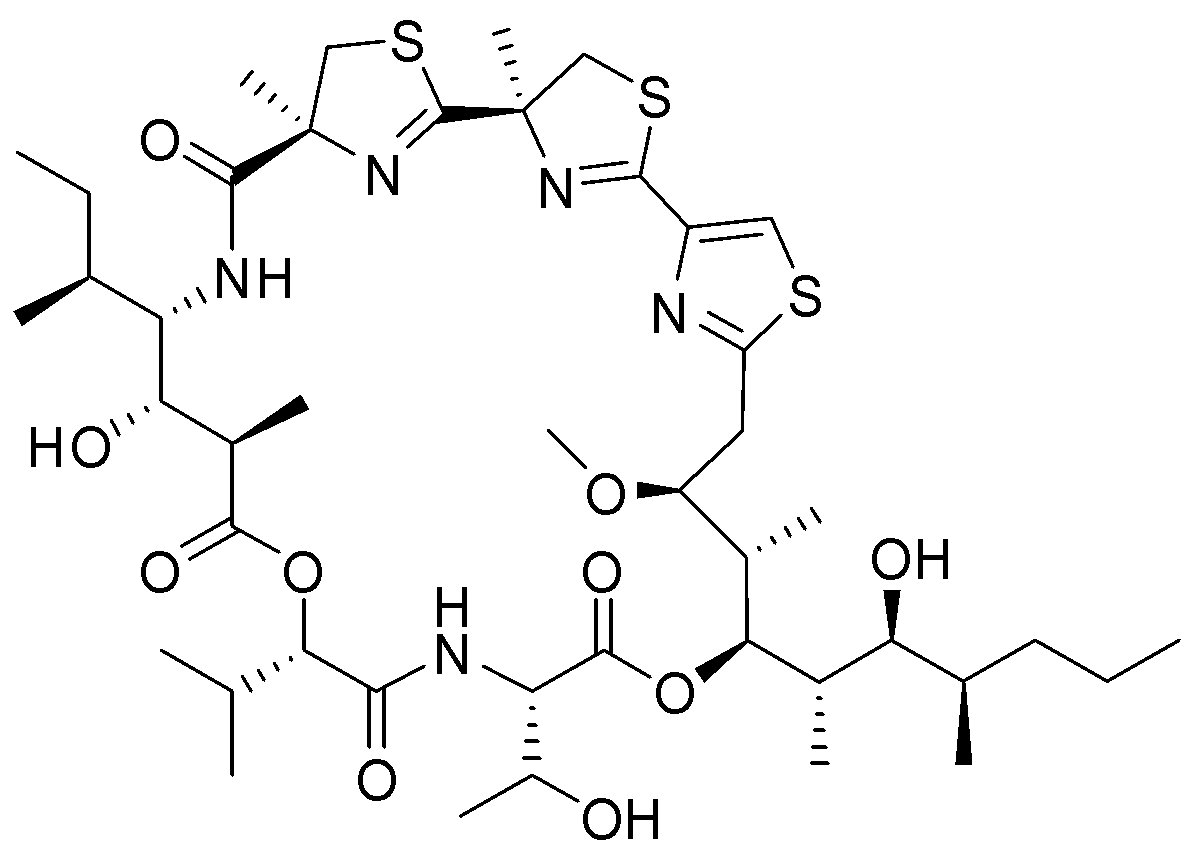 | ([Pereira, et al., 2009](#_3whwml4)) | 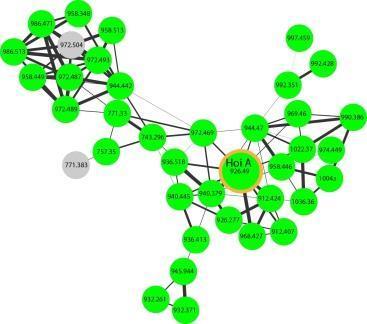 |
| 13 | Hoiamide B  [M+Na]+ | 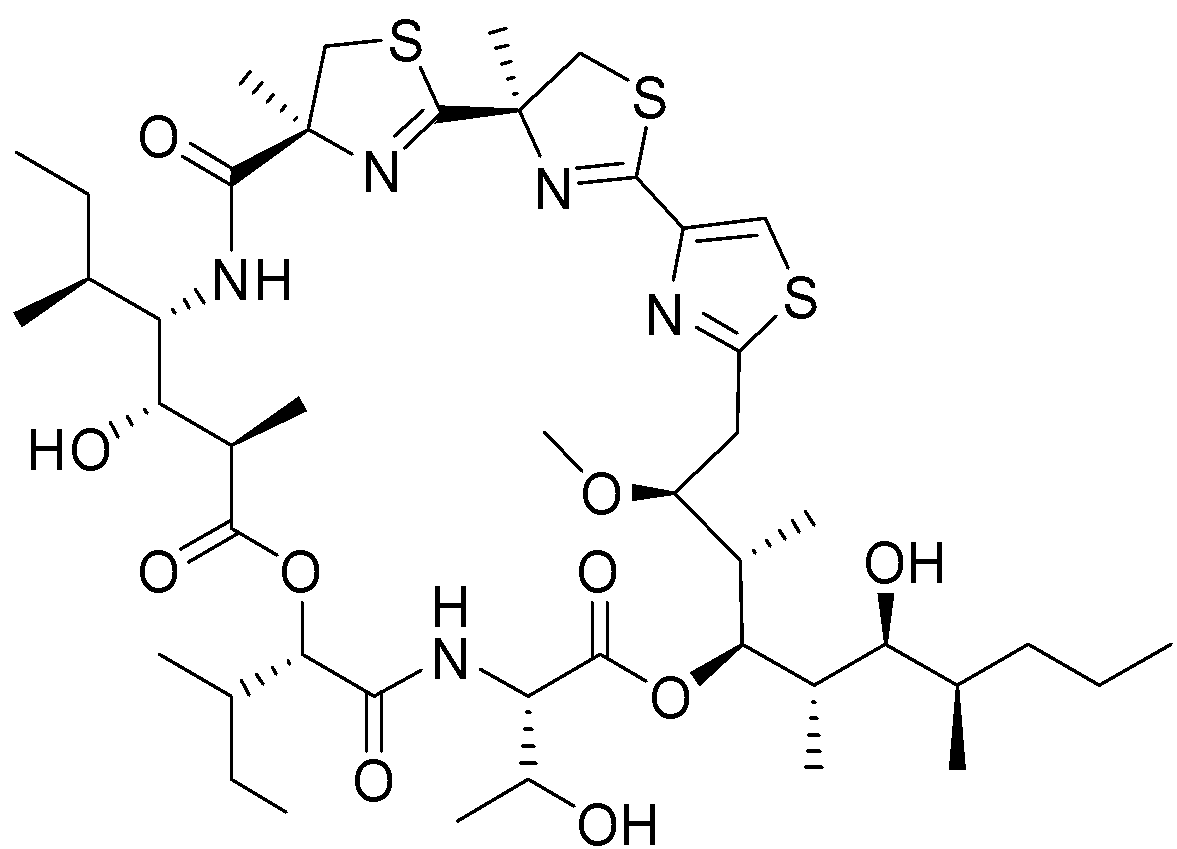 | ([Choi, et al., 2010](#_4d34og8)) | 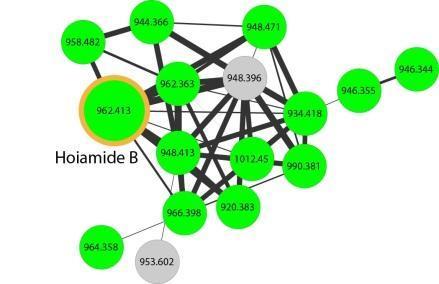 |
| 14 | Janthielamide | 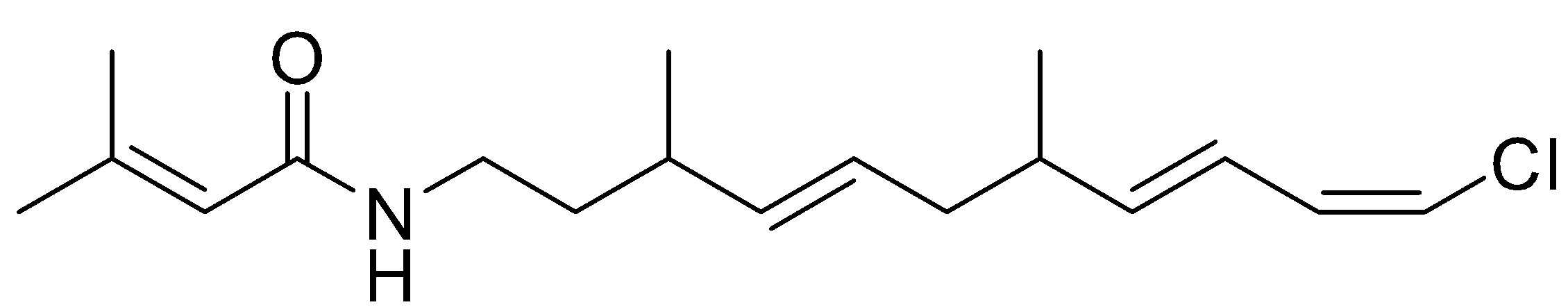 | ([Nunnery, et al., 2012](#_1y810tw)) | 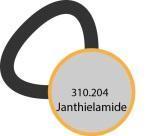 |
| 15 | Lyngbyatoxin A | 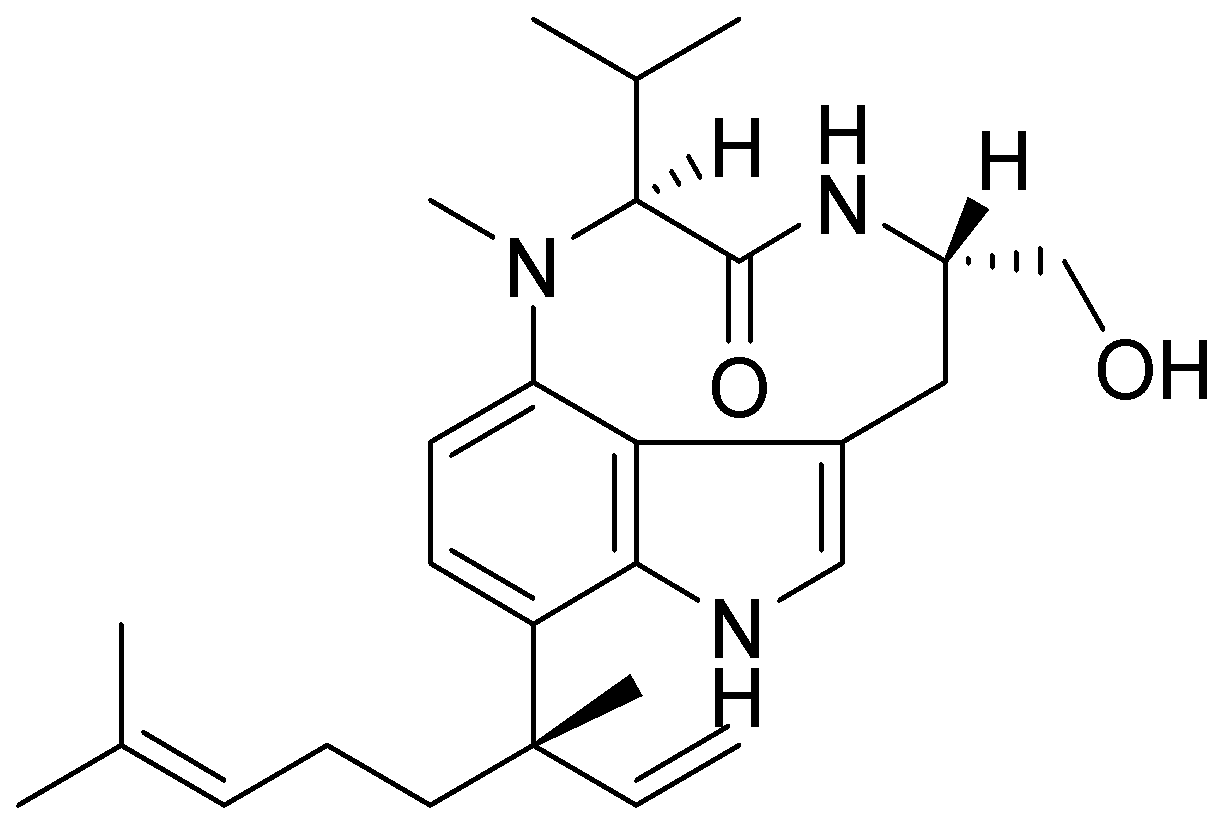 | ([Cardellina, et al., 1979](#_1t3h5sf)) | 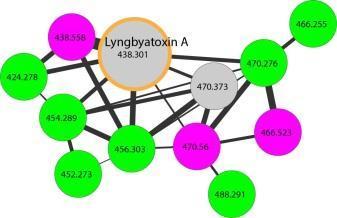 |
| 16 | Majusculamide A/B | 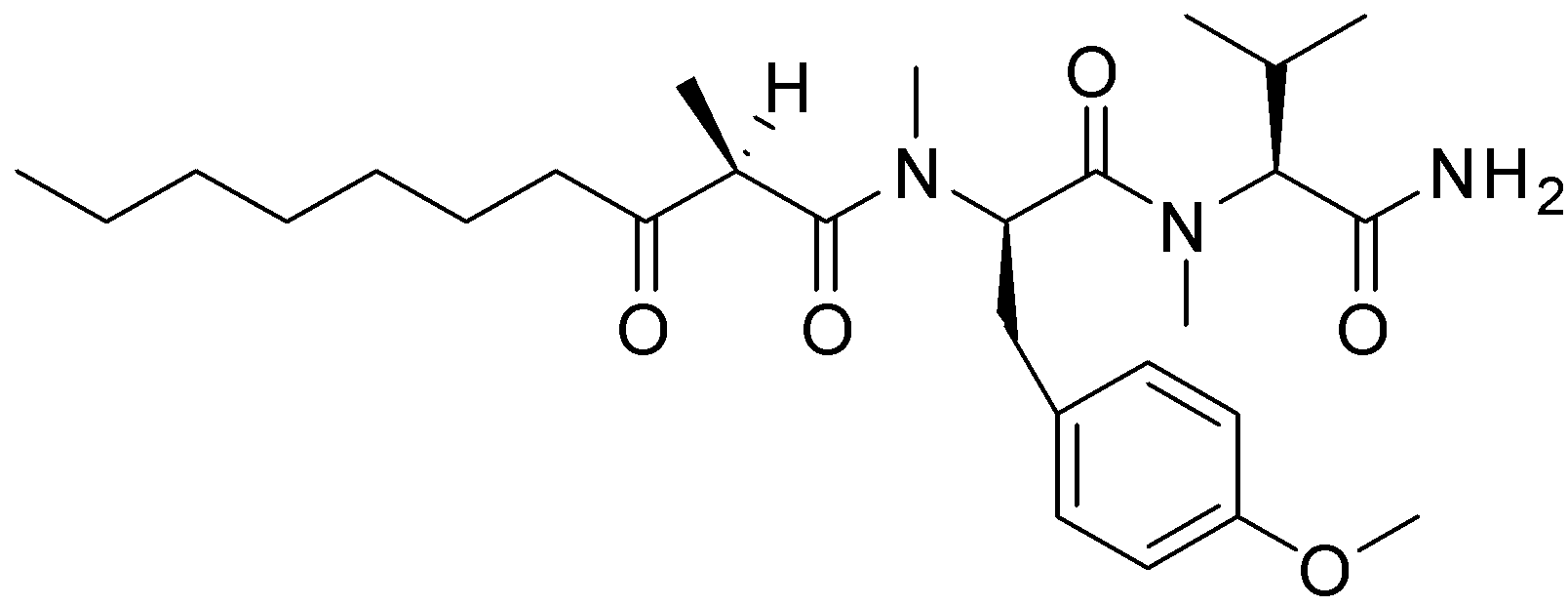 | ([Tan, et al., 2010](#_1pxezwc)) | 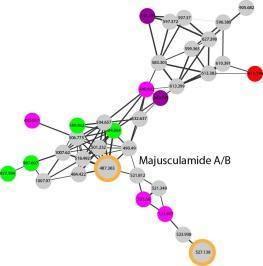 |
| 17 | Malyngamide C | 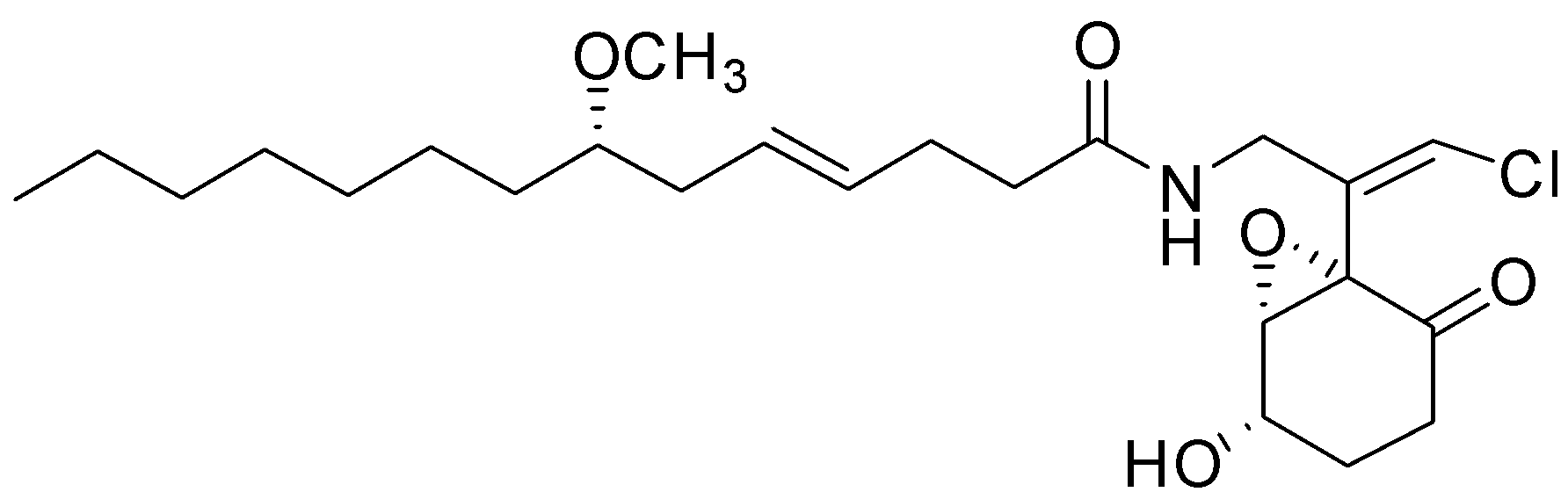 | ([Gross, et al., 2010](#_lnxbz9)) | 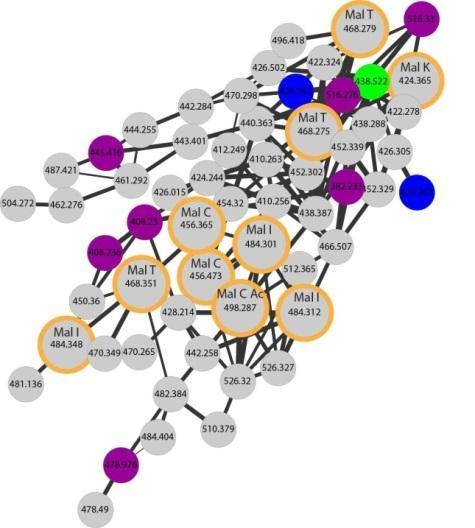 |
| 18 | Malyngamide C acetate | 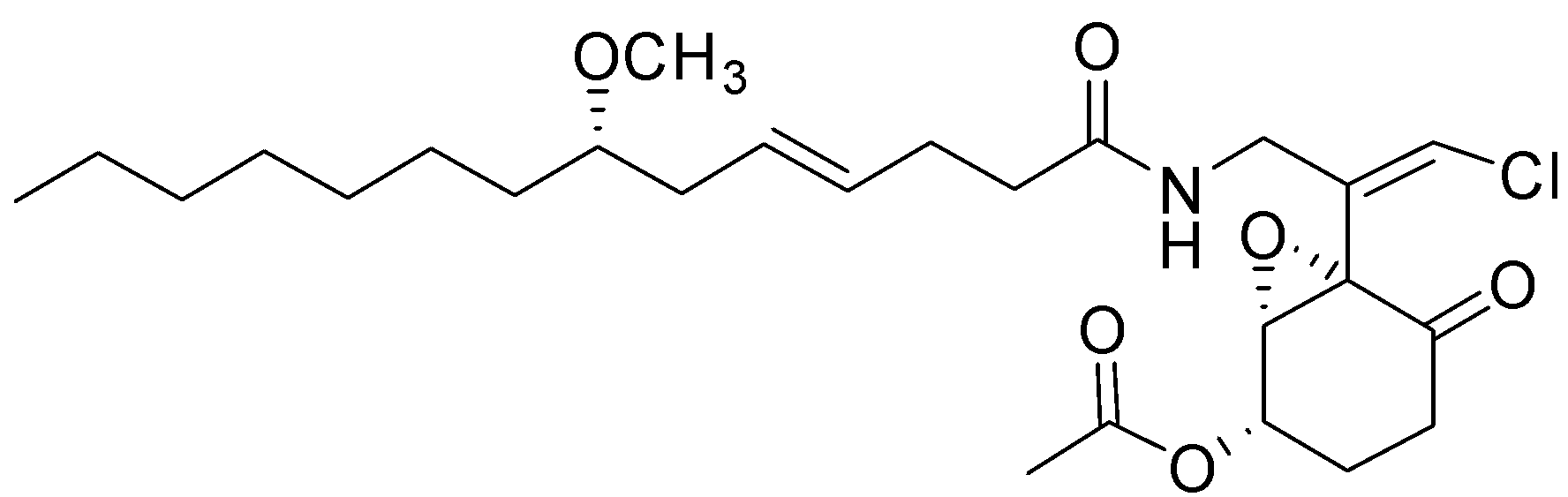 |  |  |
| 19 | Malyngamide I | 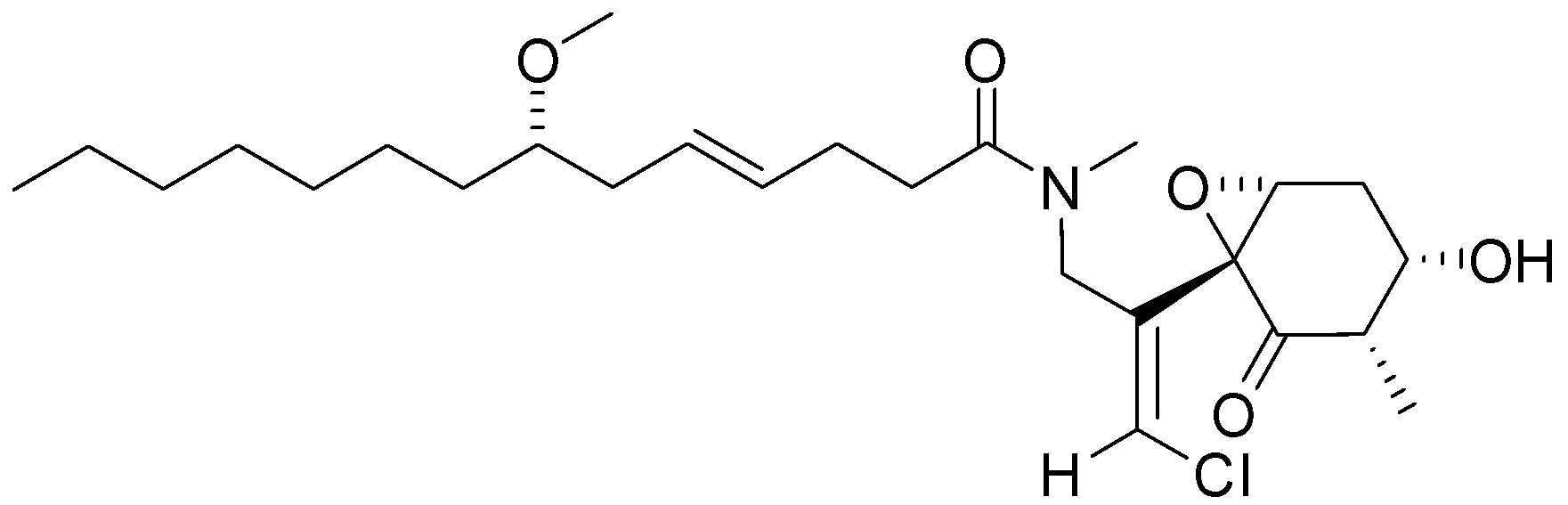 |  |  |
| 20 | Malyngamide H | 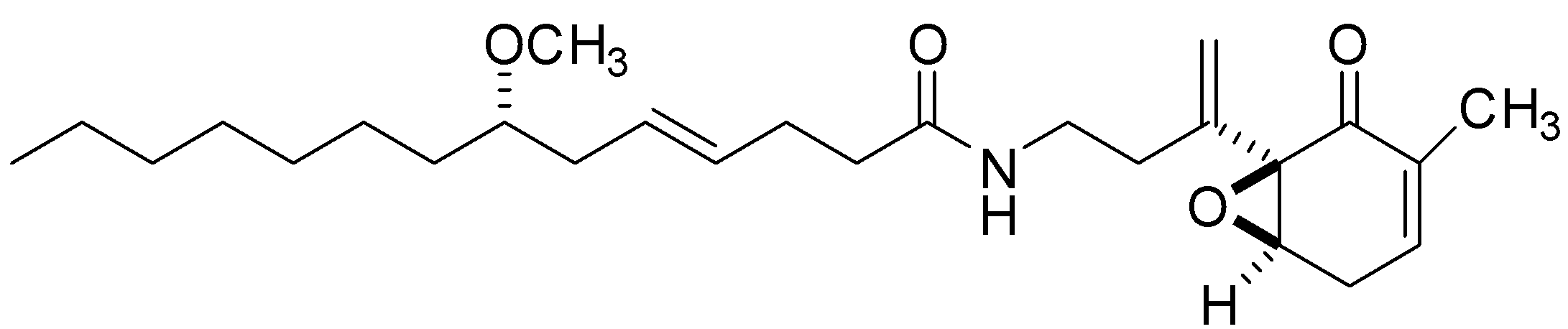 | ([Orjala, et al., 1995](#_1ci93xb)) |  |
| 21 | Malyngamide K | 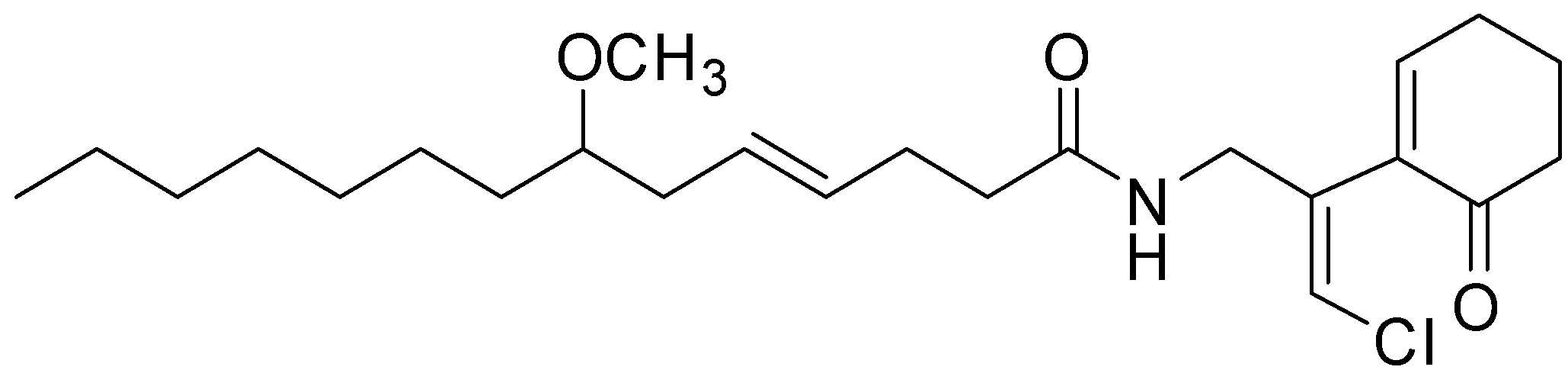 |  |  |
| 22 | Malyngamide T | 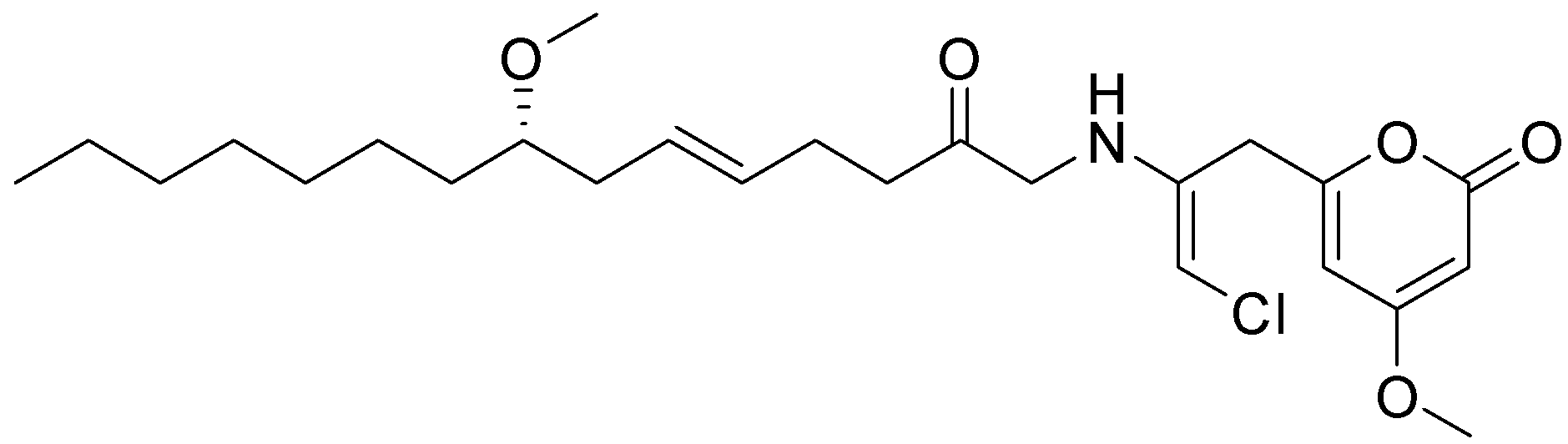 |  |  |
| 23 | Microcolin B | 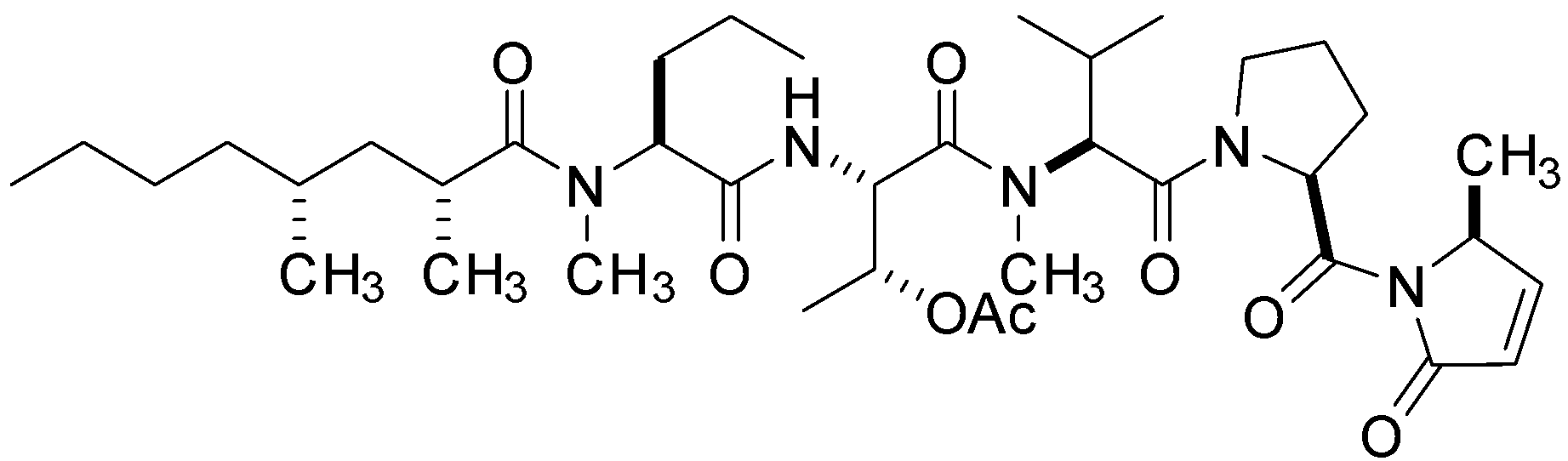 |  | 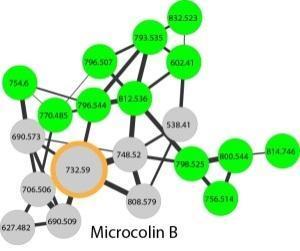 |
| 24 | Palmyramide A | 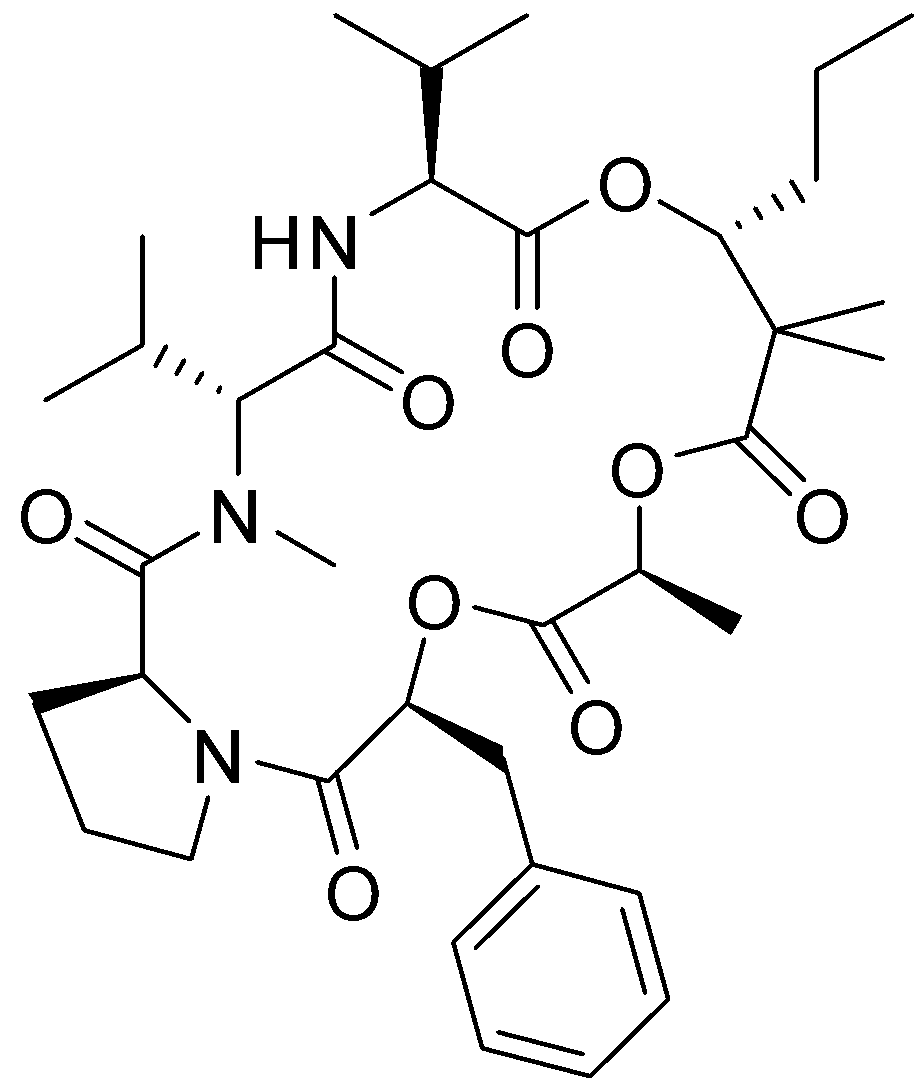 | ([Taniguchi, et al., 2010](#_2p2csry)) | 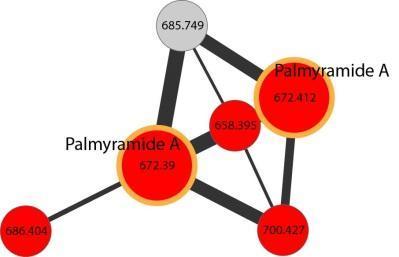 |
| 25 | Palmyrolide A | 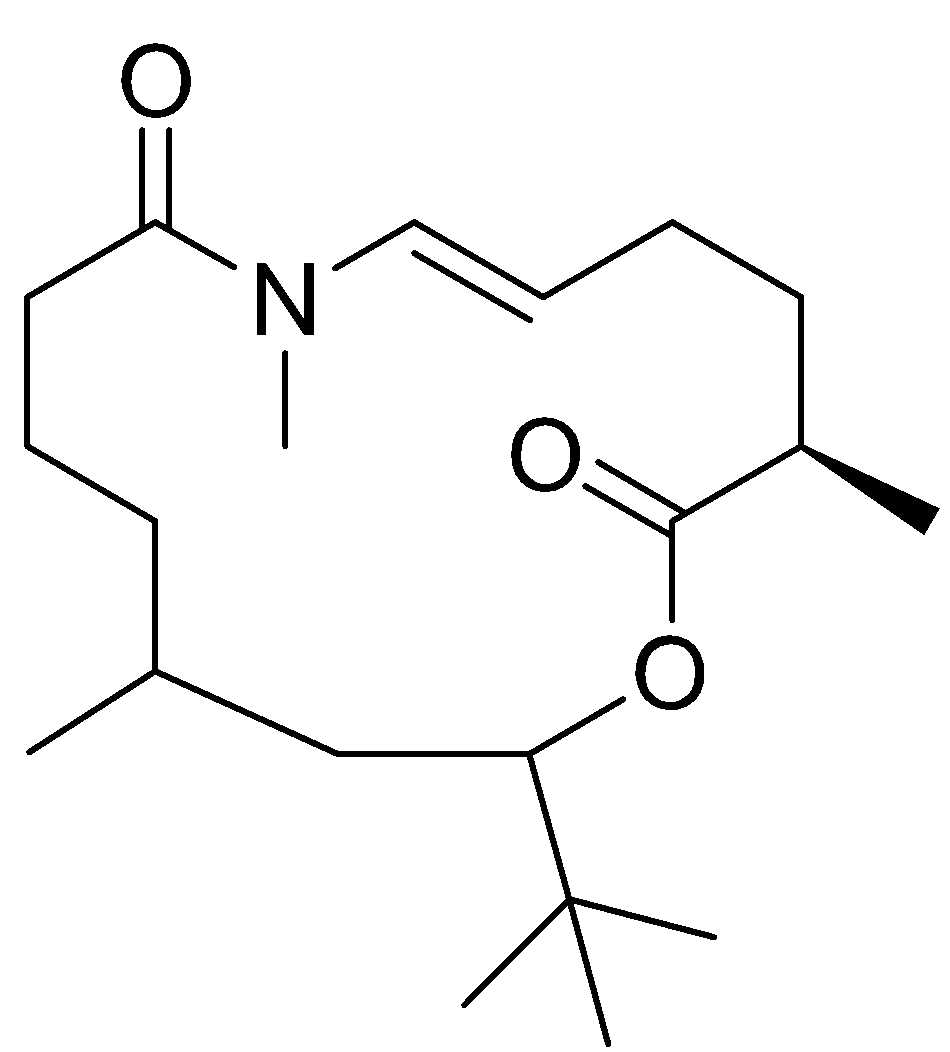 | ([Pereira, et al., 2010](#_2bn6wsx)) | 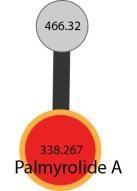 |
| 26 | Scytonemin | 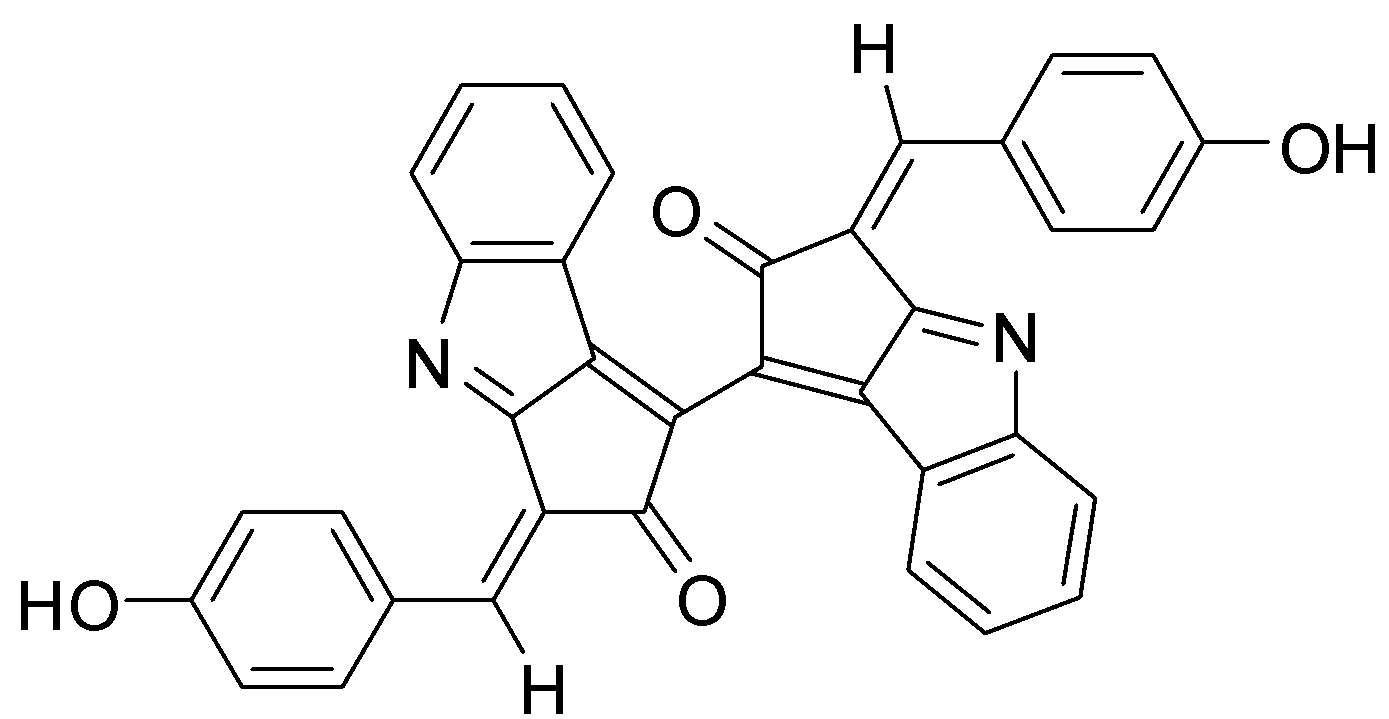 | ([Garcia-Pichel, et al., 1992](#_3rdcrjn)) | 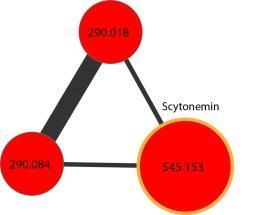 |
| 27 | Stypoltrione | 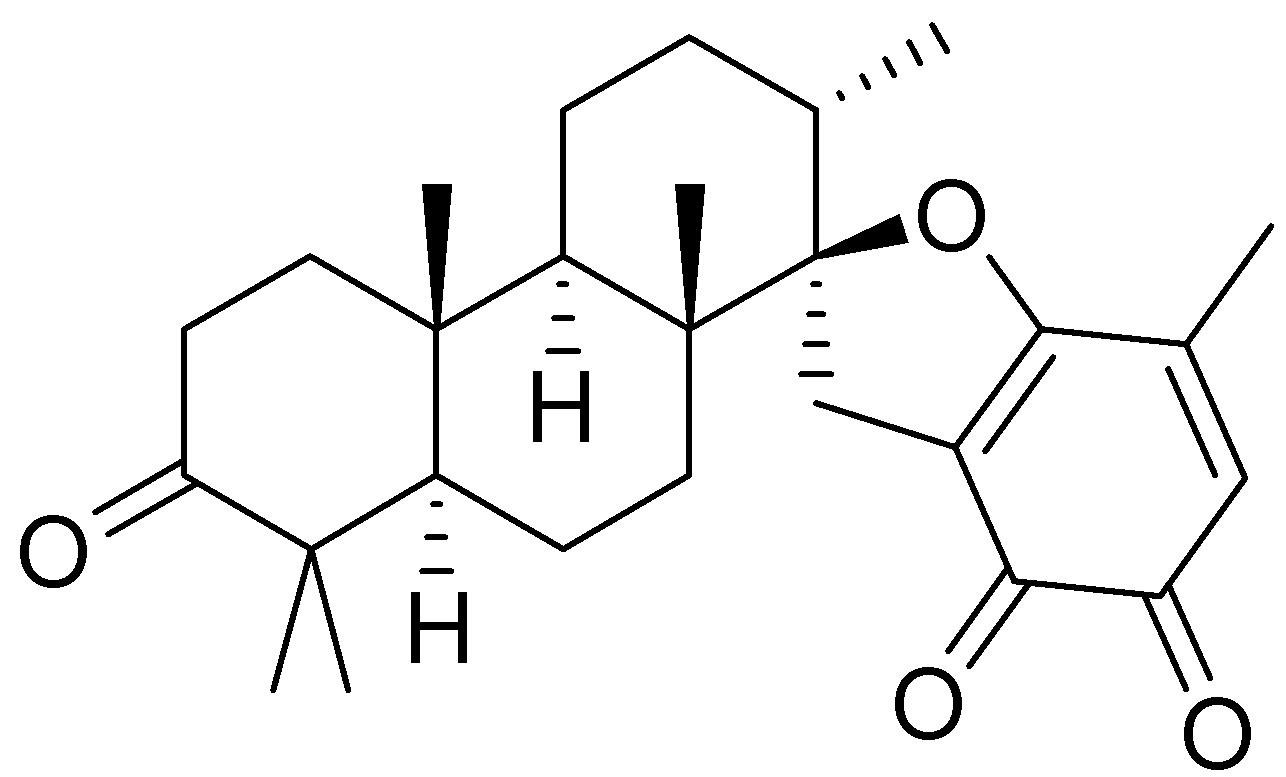 | ([O'Brien, et al., 1983](#_4i7ojhp)) | 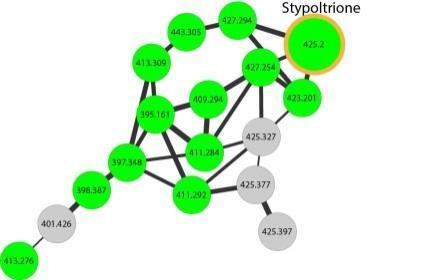 |
| 28 | Tumonoic Acid I | 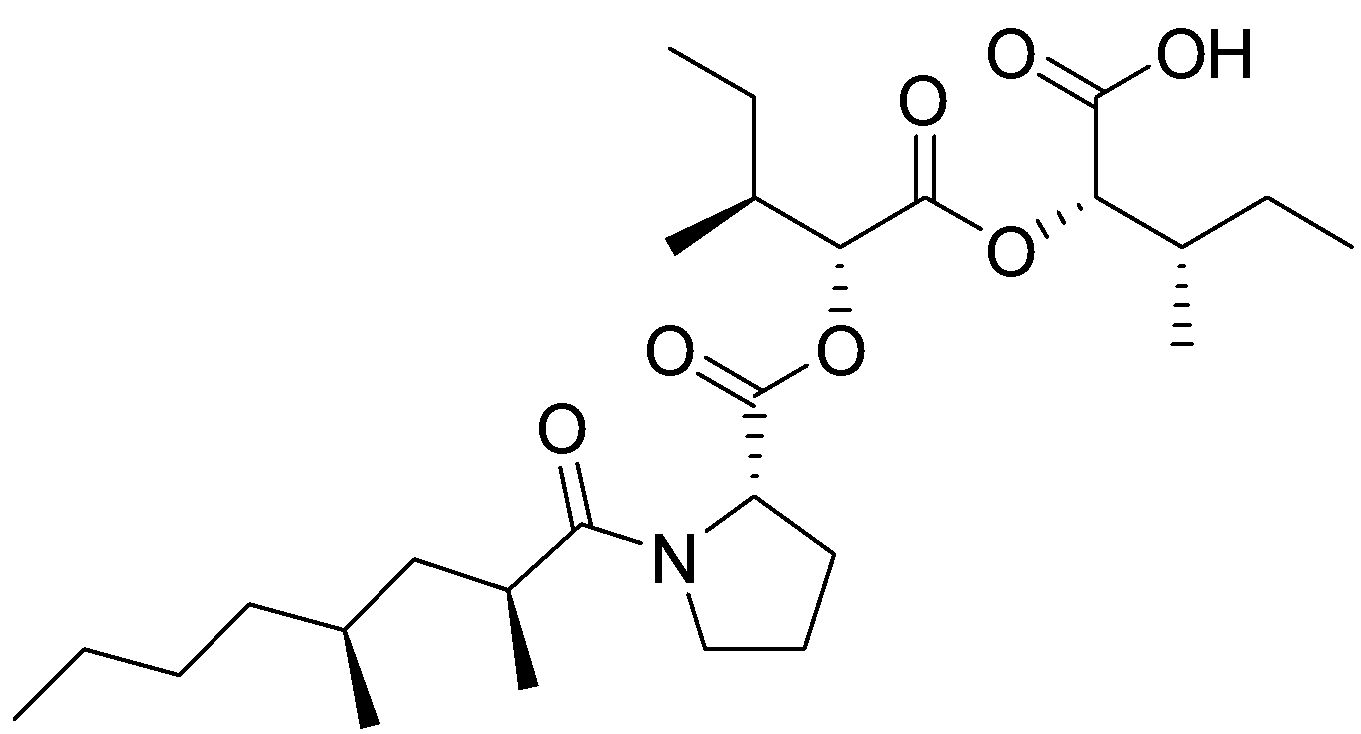 | ([Harrigan, et al., 1999](#_1ksv4uv)) | 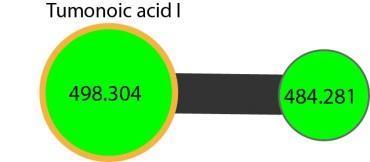 |
| 29 | Viequeamide A | 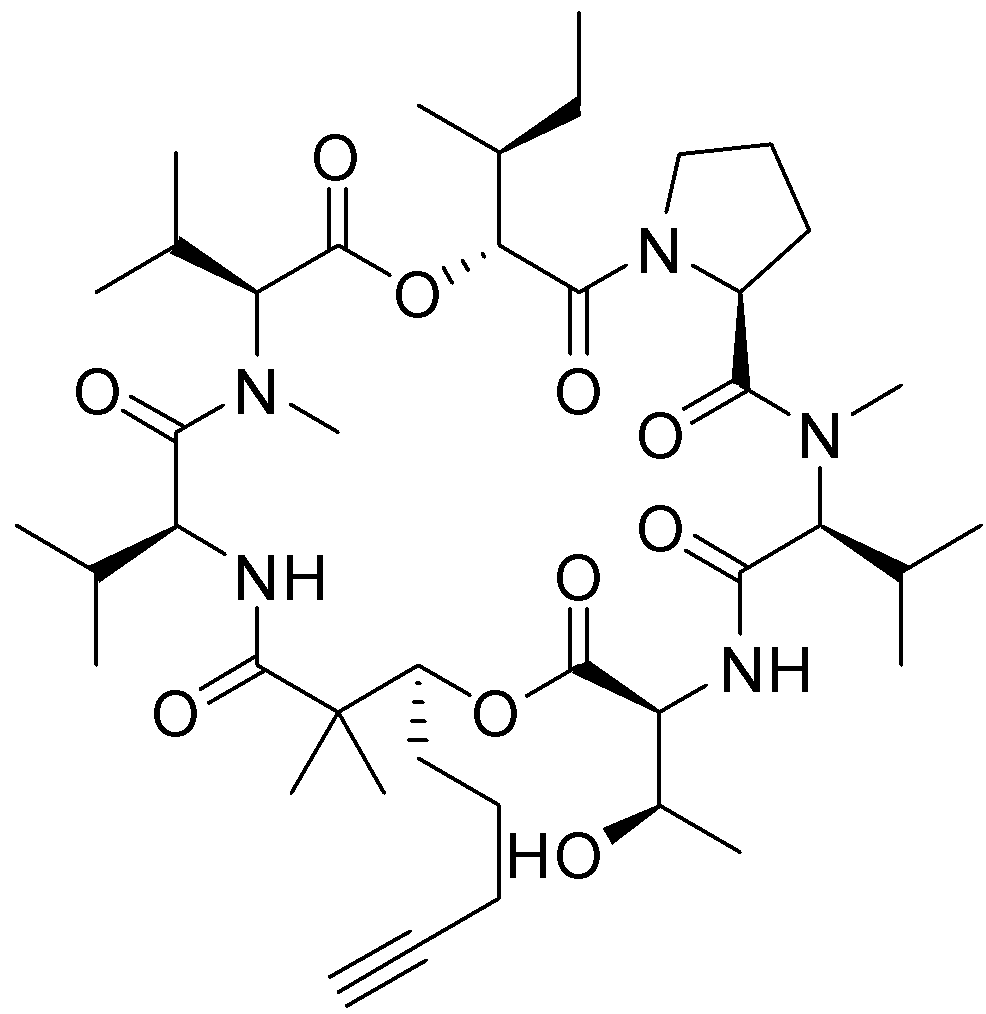 | ([Boudreau, et al., 2012](#_3dy6vkm)) | 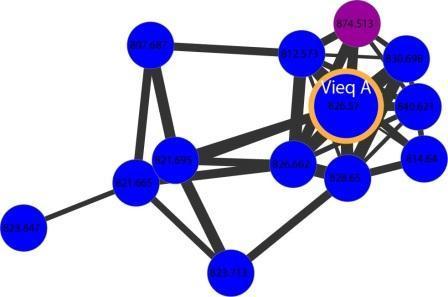 |
| 30 | Viequeamide B | 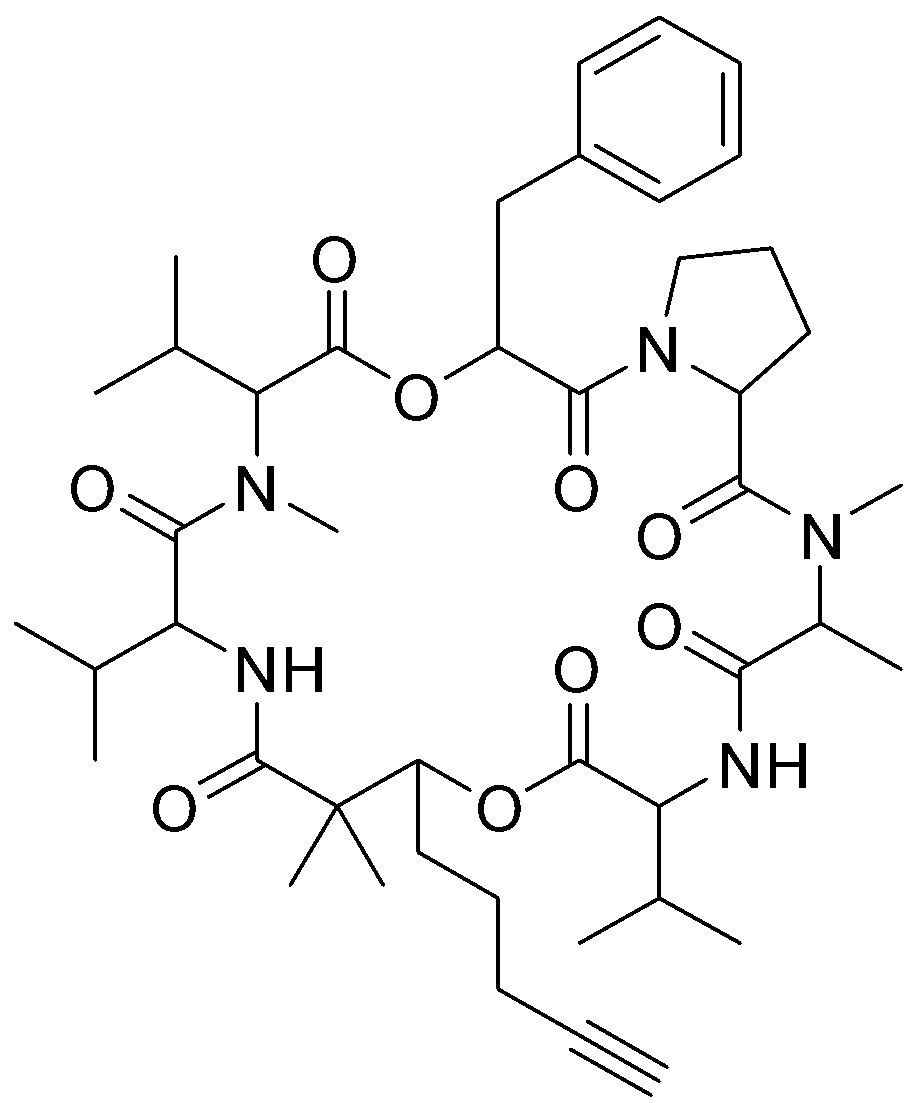 | ([Boudreau, et al., 2012](#_3dy6vkm)) | 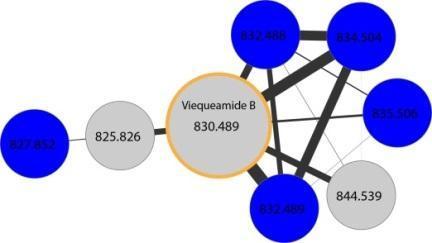 |

Reference Details:

Cardellina JH, Marner FJ, Moore RE. 1979. Seaweed dermatitis: structure of lyngbyatoxin A. *Science* **204**:193–195. PubMed: 107586 DOI: 10.1126/science.107586

Choi H, Pereira AR, Cao Z, Shuman CF, Engene N, Byrum T, Matainaho T, Murray TF, Mangoni A, Gerwick WH. 2010. The hoiamides, structurally intriguing neurotoxic lipopeptides from Papua New Guinea marine cyanobacteria. *Journal of Natural Products* **73**:1411–1421. PubMed: 20687534 DOI: 10.1021/np100468n

Esquenazi E, Coates C, Simmons L, Gonzalez D, Gerwick WH, Dorrestein PC. 2008. Visualizing the spatial distribution of secondary metabolites produced by marine cyanobacteria and sponges via MALDI-TOF imaging. *Molecular BioSystems* **4**:562–570. R21 PubMed: 18493654 DOI: 10.1039/b720018h

Garcia-Pichel F, Sherry ND, Castenholz RW. 1992. Evidence for an ultraviolet sunscreen role of the extracellular pigment scytonemin in the terrestrial Cyanobacterium chlorogloeopsis sp. *Photochemistry and Photobiology* **56**:17–23. PubMed: 1508978 DOI: 10.1111/j.1751-1097.1992.tb09596.x

Harrigan GG, Luesch H, Yoshida WY, Moore RE, Nagle DG, Biggs J, Park PU, Paul VJ. 1999. Tumonoic acids, novel metabolites from a cyanobacterial assemblage of Lyngbya majuscula and Schizothrix calcicola. *Journal of Natural Products* **62**:464–467. PubMed: 10096859 DOI: 10.1021/np980460u

Harrigan GG, Yoshida WY, Moore RE, Nagle DG, Park PU, Biggs J, Paul VJ, Mooberry SL, Corbett TH, Valeriote FA. 1998. Isolation, structure determination, and biological activity of dolastatin 12 and lyngbyastatin 1 from Lyngbya majuscula/Schizothrix calcicola cyanobacterial assemblages. *Journal of Natural Products* **61**:1221–1225. PubMed: 9784156 DOI: 10.1021/np9801211

Hooper GJ, Orjala J, Schatzman RC, Gerwick WH. 1998. Carmabins A and B, new lipopeptides from the Caribbean Cyanobacterium lyngbya majuscula. *Journal of Natural Products* **61**:529–533. PubMed: 9584405 DOI: 10.1021/np970443p

Marquez BL, Watts KS, Yokochi A, Roberts MA, Verdier-Pinard P, Jimenez JI, Hamel E, Scheuer PJ, Gerwick WH. 2002. Structure and absolute stereochemistry of hectochlorin, a potent stimulator of actin assembly. *Journal of Natural Products* **65**:866–871. PubMed: 12088429 DOI: 10.1021/np0106283

Nunnery JK, Engene N, Byrum T, Cao Z, Jabba SV, Pereira AR, Matainaho T, Murray TF, Gerwick WH. 2012. Biosynthetically intriguing chlorinated lipophilic metabolites from geographically distant tropical marine cyanobacteria. *The Journal of Organic Chemistry* **77**:4198–4208. PubMed: 22489775 DOI: 10.1021/jo300160e

Orjala J, Nagle D, Gerwick WH. 1995. Malyngamide H, an ichthyotoxic amide possessing a new carbon skeleton from the Caribbean Cyanobacterium lyngbya majuscula. *Journal of Natural Products* **58**:764–768. PubMed: 7623050 DOI: 10.1021/np50119a019

Pereira AR, Cao Z, Engene N, Soria-Mercado IE, Murray TF, Gerwick WH. 2010a. Palmyrolide A, an unusually stabilized neuroactive macrolide from Palmyra atoll cyanobacteria. *Organic Letters***12**:4490–4493. PubMed: 20845912 DOI: 10.1021/ol101752n

Pereira A, Cao Z, Murray TF, Gerwick WH. 2009. Hoiamide a, a sodium channel activator of unusual architecture from a consortium of two papua new Guinea cyanobacteria. *Chemistry & Biology* **16**:893–906. PubMed: 19716479 DOI: 10.1016/j.chembiol.2009.06.012

Tan LT, Okino T, Gerwick WH. 2013. Bouillonamide: a mixed polyketide-peptide cytotoxin from the marine Cyanobacterium moorea bouillonii. *Marine Drugs* **11**:3015–3024. PubMed: 23966034 DOI: 10.3390/md11083015

Taniguchi M, Nunnery JK, Engene N, Esquenazi E, Byrum T, Dorrestein PC, Gerwick WH. 2010. Palmyramide A, a cyclic depsipeptide from a Palmyra atoll collection of the marine Cyanobacterium lyngbya majuscula. *Journal of Natural Products* **73**:393–398. PubMed: 19839606 DOI: 10.1021/np900428h
