# Supplementary material for: Digitizing mass spectrometry data to explore the chemical diversity and distribution of marine cyanobacteria and algae
Source: eLife. 2017 May 11;6:e24214. doi: 10.7554/eLife.24214 (PMC5441867; doi:10.7554/eLife.24214)
Supplement: Supplementary file 3. — 13C and 1H chemical shifts were determined by HSQC and HMBC spectra. Hiv = 2 hydroxyisovaleric acid (600 MHz for 1H, 125 for 13C), the solvent (DMSO-d6) and the temperature (298K). DOI: http://dx.doi.org/10.7554/eLife.24214.017 [file elife-24214-supp3.docx]

| Residue | Position | δ_C_*, type | δ_H_, mult. (*J* in Hz) |
| --- | --- | --- | --- |
| Gly | CO | 168.6, C | - |
|  | NH | - | 7.33, dd (6.6, 3.5) |
|  | α | 41.5, CH_2_ | 4.04, dd (17.5, 6.7)  3.76, dd (15.5, 3.4) |
| Ile | CO | 170.2, C | - |
|  | NH | - | 7.90, d (9.3) |
|  | α | 56.7, CH | 4.31, dd (9.3, 5.8) |
|  | β | 35.4, CH | 1.96, m |
|  | γ_1_ | 24.1, CH_2_ | 1.49, m  1.13, m |
|  | γ_2_ | 15.6, CH_3_ | 0.86, d (6.8) |
|  | δ | 11.1, CH_3_ | 0.81, t (7.4) |
| Hiv | CO | 169.4, C | - |
|  | α | 77.8, CH | 5.04, d (2.9) |
|  | β | 29.4, CH | 2.37, m |
|  | γ_1_ | 19.0, CH_3_ | 0.95, m |
|  | γ_2_ | 16.0, CH_3_ | 0.90, d (6.9) |
| Val | CO | 172.8, C | - |
|  | NH | - | 7.99, d (4.5) |
|  | α | 61.0, CH | 3.88, dd (8.7, 4.5) |
|  | β | 27.8, CH | 2.15, m |
|  | γ_1_ | 18.9, CH_3_ | 0.96, m |
|  | γ_2_ | 19.9, CH_3_ | 1.03, d (6.6) |
| Dhoya | 1 | 174.8, C | - |
|  | 2 | 45.6, C | - |
|  | 3 | 78.9, CH | 4.71, d (11.3) |
|  | 4 | 24.8, CH_2_ | 1.95, m  1.65, m |
|  | 5 | 24.5, CH_2_ | 1.42, m  1.30, m |
|  | 6 | 17.0, CH_2_ | 2.14, m |
|  | 7 | 84.5, C |  |
|  | 8 | 71.7, CH | 2.78, s |
|  | 9 | 24.1, CH_3_ | 0.96, m |
|  | 10 | 20.5, CH_3_ | 1.29, m |

**Figure 5-Supplementary file 3. 2D NMR spectroscopic data for amino acids residues of yuvalamide A.** ^13^C and ^1^H chemical shifts were determined by HSQC and HMBC spectra. Hiv = 2-hydroxyisovaleric acid (600 MHz for ^1^H, 125 for ^13^C), the solvent (DMSO-d6) and the temperature (298K).
